# Antibody Responses to Transglutaminase 3 in Dermatitis Herpetiformis: Lessons from Celiac Disease

**DOI:** 10.3390/ijms23062910

**Published:** 2022-03-08

**Authors:** Helka Kaunisto, Teea Salmi, Katri Lindfors, Esko Kemppainen

**Affiliations:** 1 Celiac Disease Research Center, Faculty of Medicine and Health Technology, Tampere University, FI-33520 Tampere, Finland; helka.kaunisto@tuni.fi (H.K.); teea.salmi@tuni.fi (T.S.); katri.lindfors@tuni.fi (K.L.); 2 Department of Dermatology, Tampere University Hospital, FI-33520 Tampere, Finland

**Keywords:** dermatitis herpetiformis, celiac disease, transglutaminase 3

## Abstract

Dermatitis herpetiformis (DH) is the skin manifestation of celiac disease, presenting with a blistering rash typically on the knees, elbows, buttocks and scalp. In both DH and celiac disease, exposure to dietary gluten triggers a cascade of events resulting in the production of autoantibodies against the transglutaminase (TG) enzyme, mainly TG2 but often also TG3. The latter is considered to be the primary autoantigen in DH. The dynamics of the development of the TG2-targeted autoimmune response have been studied in depth in celiac disease, but the immunological process underlying DH pathophysiology is incompletely understood. Part of this process is the occurrence of granular deposits of IgA and TG3 in the perilesional skin. While this serves as the primary diagnostic finding in DH, the role of these immunocomplexes in the pathogenesis is unknown. Intriguingly, even though gluten-intolerance likely develops initially in a similar manner in both DH and celiac disease, after the onset of the disease, its manifestations differ widely.

## 1. Introduction

Dermatitis herpetiformis (DH) is an extraintestinal manifestation of celiac disease (CeD). Both conditions are driven by the ingestion of dietary gluten in wheat, rye and barley, which induces an inflammatory response featuring B and T cell activation. While CeD and DH patients both evince small intestinal inflammation and often also villous atrophy, CeD patients suffer primarily from gastrointestinal symptoms, whereas DH manifests additionally, or exclusively, with a blistering rash, most often affecting the elbows, knees and buttocks. The primary diagnostic finding in DH is the appearance of granular deposits of immunoglobulin A (IgA) in the papillary dermis, particularly in the perilesional areas of the skin [1].

Irrespective of the different primary manifestations, DH and CeD share genetic susceptibility conferred by *HLA-DQ2* or *-DQ8* [2]. The majority of untreated CeD patients are seropositive for antibodies against gluten-derived peptides and transglutaminase 2 (TG2), a member of the transglutaminase family of enzymes and the primary autoantigen in CeD [3]. Likewise, most DH patients develop circulating TG2 autoantibodies [4]. Approximately one-third of CeD patients are also seropositive for autoantibodies against transglutaminase 3 (TG3). Meanwhile, a much higher proportion of DH patients develop circulating autoantibodies against TG3, which is considered to be the primary autoantigen in this phenotype [5]. Similar to CeD, circulating autoantibodies against both TG2 and TG3 disappear as a result of gluten-free diet (GFD), the treatment of choice for DH. The granular immunocomplexes in the dermis, considered to comprise TG3 and IgA-class antibodies against TG3, may persist in the skin of seronegative patients for months or even years after the initiation of GFD [5,6].

In this review, we discuss the immunological processes relevant for TG3 autoantibody response and potentially underlying DH disease pathogenesis.

## 2. Transglutaminase 3-The Epidermal Transglutaminase

Transglutaminases constitute a family of nine enzymes which crosslink proteins covalently in a calcium (Ca2+)-dependent manner. TG3 is expressed as an inactive 77 kDa zymogen which must be activated by limited proteolytic processing into two fragments (44 kDa and 30 kDa) of which the larger, N-terminal fragment carries the catalytic activity [7,8]. The enzyme responsible for this processing has not been identified but it has been suggested that at least cathepsin L released from degraded lysosomes could cleave the TG3 zymogen in vivo [9]. In vitro studies have shown that proteinase K, trypsin, dispase and thrombin are also able to activate TG3 via cleavage [8,10].

**Figure 1:**
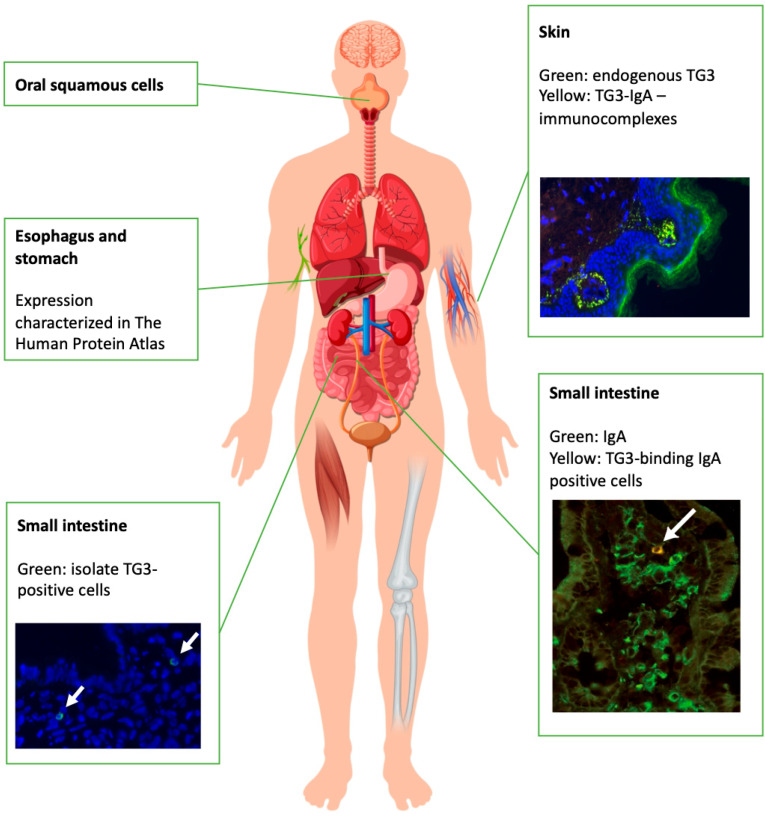
Known sites of human TG3 expression (left panel) and reported sites of immunological responses against TG3 (right panel) [15,16].

Once activated, TG3 catalyzes the formation of isopeptide bonds between the γ-carboxamide group of glutamine and the ε-amino group of lysine via an enzyme-substrate thioester intermediate. TG3 is best known for its role in the formation of the cornified envelope, linking differentiated keratinocytes and inner hair sheath cells (Figure 1). Accordingly, TG3 protein expression was first discovered in hair follicles [11,12] and later in the epidermis, brain, stomach, spleen, small intestine, testes, and skeletal muscles [13,14]. Although the expression of TG3 has been detected in a number of tissues and organs, its biological function has only been well-described for skin, where it is expressed predominantly in the stratified squamous epithelium and has not been thoroughly investigated in other tissues or organs.

TG3 has been linked to gluten-sensitive autoimmune disorders together with two other transglutaminases: TG2 and TG6. All three transglutaminases are encoded by genes located on chromosome 10q21 and share significant sequence homology, particularly with respect to the catalytic domain. Likewise, all three enzymes are able to deamidate gluten-derived gliadin peptides, although with isoform-dependent efficiency and substrate specificity [17]. These enzymes also differ with respect to their ability to form covalent iso-peptide complexes with gluten. TG2 can form complexes with gliadin peptides via both iso-peptide and thioester bonds. In comparison, TG3 and TG6 can form enzyme–peptide thioester complexes less efficiently and TG3 lacks the ability to form iso-peptide-linked complexes with gliadin.

## 3. Systemic Responses against TG3 in DH

DH patients typically produce autoantibodies against TG3 in a gluten-dependent manner. DH is often considered to develop as a result of prolonged gluten exposure and untreated CeD, but it is not known whether the autoimmune responses against TG2 and TG3 develop in certain patients in parallel, or whether TG3 merely becomes targeted via gradual loss of antigen specificity against TG2 in a subset of CeD patients. It is noteworthy, however, that while both conditions respond to gluten-free diet (GFD), if gluten is reintroduced to the diet of DH patients, the disease may manifest with either gastrointestinal or skin symptoms. The latter response would suggest that a certain component in the permanent loss of immune tolerance is very specific to DH. We have reviewed the current understanding—or lack thereof—of the immunological processes potentially underlying the development of gluten-driven TG3 autoimmunity.

### 3.1. Mechanisms of Anti-TG3 Antibody Development

It is unclear why some patients with CeD develop antibodies towards TG3, and why only a subset of these TG3-antibody positive subjects have DH. In addition, how TG2 and TG3 antibody responses develop from an initial antigliadin response has for long remained unknown. However, recent advances in CeD research have suggested that anti-TG2 responses arise as a result of epitope spreading from gliadin to TG2, mediated by anti-TG2 B cells interacting with antigliadin T cells. Epitope spreading generally refers to the process where an immune response develops towards an epitope distinct from the original, disease-causing epitope [18]. Epitope spreading is well-characterized in antibody-mediated diseases such as systemic lupus erythematosus [19]. While less literature is available on the development of TG3 antibody responses, it has been proposed that TG3 antibodies originate from TG2 antibodies, as evidenced by a degree of antibody cross-reactivity in a subset of DH patients [5]. This is also supported by the fact that TG3 antibodies are rarely detected in children with CeD, in contrast to TG2 antibodies [20]. Furthermore, autoantibodies against TG2, TG3 and TG6 have been implicated in gluten-linked autoimmune disorders, implying potential overlap between the specific autoimmune responses. However, it is also possible that TG3 antibodies arise similarly to TG2 antibodies as a result of epitope spreading from gliadin. Before addressing the possible mechanisms for TG3 antibody development in more detail, we are going to briefly review the development of anti-TG2 antibody response using TG2 in CeD as an example.

Although there are a few articles suggesting that TG2 antibody responses are enabled by T cells recognizing TG2 [21,22], the existence of these cells has remained enigmatic [23]. Due to this discrepancy, the development of IgA class-switched TG2 antibodies has puzzled CeD researchers. The most obvious explanation would be that anti-TG2 responses are T cell independent. This would imply that either TG2-specific B cells are B1 (T cell independent) B cells, or that TG2 is able to function as a thymus-independent antigen. The TG2 antibodies characterized from CeD patients are typically class-switched to an IgA isotype, as well as having gone through affinity maturation [24,25,26]. Both the aforementioned processes occur at very low levels in B1 B cells and are conventionally considered to require T cell help, which makes it unlikely that TG2-specific B cells in CeD and DH are B1 B cells. If TG2 were to act as a thymus-independent antigen, it could activate B cells to produce antibodies without T cell help. However, thymus-independent antigens have to possess strong crosslinking properties, such as bacterial polysaccharides that contain highly repetitive structures. TG2 does not contain such structures, and it has been found that anti-TG2 antibodies bind specific epitopes in the N-terminal region of TG2 [27,28]. It has been hypothesized that since TG2 remains enzymatically active while bound to B cell receptors (BCRs), it could crosslink B cell receptors and thereby activate these cells [24]. In addition to this, it has been shown that B cells are also able to bind multimers of several TG2 molecules complexed with gliadins [29,30], which could also lead to increased crosslinking of B cell receptors [29]. Although BCR crosslinking antigens can activate B cells without T cell help, in autoreactive cells, this type of recognition of strongly crosslinked antigens conventionally leads to clonal deletion [31]. It is therefore unlikely that TG2 crosslinks BCRs sufficiently to lead to T cell independent activation. It has also been shown in vitro that TG2-specific B cells have markedly reduced proliferation in response to TG2 if T cell help is unavailable [29]. Further supporting T-cell-dependence is the HLA-dependency of both CeD and DH. All of these observations support the notion that anti-TG2 antibody responses require T cell help for their initiation. Although TG3 antibody responses are less well-characterized, factors such as HLA-dependency of DH [32] could indicate that TG3 antibodies in CeD and DH are also T cell-dependent.

**Figure 2:**
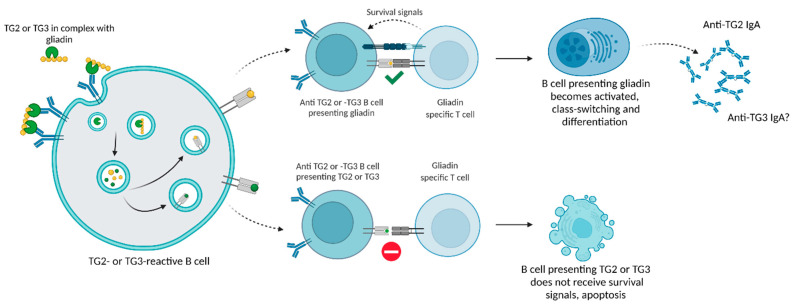
Epitope spreading from gliadin to TG2 or TG3. A simplified depiction of the suggested mechanism for epitope spreading during CeD and DH. B cells specific to TG2 and/or TG3 internalize and process gliadin–TG2 or –TG3 complexes through the endocytic pathway, leading to presentation of peptides on HLA II molecules. Gliadin-specific CD4+ T cells give survival signals to gliadin-presenting B cells, while TG2- or TG3-presenting B cells do not receive survival signals. Activated B cells class-switch into IgA and produce anti-TG2 or -TG3 antibodies. Created with BioRender.com.

Having now established that the production of TG2 antibodies most likely depends on T cell help, we still face the aforementioned dilemma that TG2- or TG3-specific T cells have not been universally recognized in CeD or DH patients. It has been proposed that TG2 antibodies arise as a result of epitope spreading from gliadin after the failure of tolerance mechanisms towards autoreactive B cells during development [33]. These B cells have been thought to be clonally ignorant after having evaded central tolerance [33]. TG2-specific B cells are present in CeD patient intestine [16,25,26,34], and when in the intestine, the TG2-autoreactive B cells are thought to bind complexes of TG2 bound to gliadin peptides [17,29,30,35,36]. After internalization, the TG2–gliadin complex becomes degraded into peptide fragments by endosomal proteases. These fragments are presented to CD4+ T cells on class II HLA molecules. The B cell does not distinguish which peptide fragment was the epitope bound by the BCR and, therefore, presents both TG2 and gliadin peptides to T cells. When gliadin-specific CD4+ T cells are presented with deaminated gliadin peptides, they become activated and, in turn, give the antigen-presenting B cells signals initiating class switching and affinity maturation. The process is illustrated in Figure 2. This type of mechanism is perhaps better known as the hapten-carrier effect, where allergy or autoimmune disease towards haptens develops as a result of complexes formed between carrier proteins and small molecule antigens [37]. The possibility of TG2-specific B cells presenting gliadin to T cells and thereafter receiving the appropriate signals for proliferation and class switching would indeed give a plausible explanation to the dilemma presented earlier. There are, however, a few prerequisites that need to be fulfilled in order for this model to function. Most importantly, gliadin–TG2 complex formation has not yet been proven in vivo in humans, despite being well-established in vitro and in mice [17,29,30,35,36]. Assuming that TG2 is indeed able to create complexes with gliadins in vivo, in order for anti-TG2 responses to develop, the tolerance mechanisms that B cells are subjected to need to fail. du Pré et al. (2019) elegantly demonstrated that TG2-specific B cells do not differ in functionality from endogenous B cells in mice, and evaded tolerance mechanisms. This study was executed by creating transgenic mice possessing TG2-specific B cell receptors derived from CeD patients [33]. Assuming that clonal ignorance was the reason for the development of TG2-reactive B cells, we should be able to find these autoreactive B cells in the general population. Finding these autoreactive B cell clones in healthy individuals would prove that TG2-reactive B cells develop endogenously. However, their identification of such cells might prove difficult before they have been clonally expanded as a result of activation. Although efforts have been made in order to ascertain how TG2 antibodies develop, knowledge on the development of TG3 responses is lacking. TG3-reactive B cell clones have not been modelled in animal studies, nor has their interaction with gliadin-specific CD4+ T cells been assessed. What we do know is that TG3 has been found to create complexes with gliadin peptides [17]. The complexes created by TG3 and gliadin in vitro are linked through a thioester bond, whereas TG2 has been found to create both iso-peptide and thioester linkages [17]. Findings of TG3 forming complexes with gliadin [17] render it plausible for us to imagine that the mechanism for anti-TG3 antibody development could be somewhat similar to that of TG2 antibody development.

A plausible model for the development of TG3 antibodies in DH could follow the mechanisms described above (Figure 2), where B cells autoreactive to TG3 evade the body’s tolerance mechanisms and develop like any other B cell. Once these B cells locate to the intestine, they internalize complexes of gliadin and TG3, and present gliadin peptides to gliadin-specific T cells. In individuals possessing the predisposing genetic background, T cells give activating signals to the B cells that presented the gliadin. In this way, epitope spreading from gliadin to TG3 allows for the development of class-switched, TG3-reactive plasma cells. This model suggests that TG3 responses arise from strictly TG3-reactive B cells, and not as a result of cross-reactivity between TG2 antibodies with TG3. Assuming B cells autoreactive to all TG isoforms in addition to TG2 and TG3 evade tolerance mechanisms in this way, we would also have an explanation as to why some DH and CeD patients have autoantibodies against TG6 [38]. This hypothesis also requires TG3 to be available to the intestinal B cells. While TG2 expression in the intestine is well-established [39], the evidence for TG3 expression in the intestine is scarce. TG3 has been found in sporadic cells in the intestine of selected DH patients via fluorescent staining [40], and anti-TG3 plasma cells have been found in approximately half of DH patients following gluten challenge, but only in one CeD patient [41]. Thus, the expression of TG3 in the intestine is low, if present at all. According to The Human Protein Atlas, TG3 is expressed in the esophagus, which opens up the possibility of TG3 shedding into the digestive track and ending up in the intestinal lumen, similarly to TG2, which is thought to shed from dying enterocytes [42], enabling the antigen to become available to B cells. TG3 could, theoretically, follow a similar pattern of release from the epithelium into the esophagus, leading to small amounts of the antigen finding their way into the intestine. It is of course entirely possible that the anti-TG3 antibody response observed in DH originates from a distinct site, and not the gastrointestinal tract. However, given the scarce literature available on DH-specific immune responses, one can only speculate the plethora of options. We have chosen to base our reasoning on the literature available on TG2 responses in CeD. Assuming that TG2 antibodies and TG3 antibodies arise from separate B cells, we would expect the production of different TG antibodies to occur at roughly the same rate. However, we mostly observe TG2 antibodies in CeD patients [20,43,44,45,46]. One explanation for this discrepancy could be antigen availability. Given that TG2 is able to create iso-peptide and thioester linkages with gliadin, while TG3 only creates thioester linkages [17], it is conceivable to imagine that TG2 is able to sequester most of the available gliadin proteins during gluten exposure as a result of more effective complex-forming abilities than TG3. This could lead to mostly anti-TG2 B cells becoming activated, unless the gluten exposure is prolonged as proposed in DH development [5,47,48], in which case, more antigen would be available for anti-TG3 B cells. As noted, TG2 is abundantly expressed in the gut while TG3 is not. This would also contribute to the restricted access of B cells to TG3.

Another possible model for TG3 antibody development assumes that TG3 antibodies originate from the cross-reactivity of TG2 antibodies with TG3. This model suggests that initial TG2 responses with weak affinity to TG3 results in the eventual development of high-affinity TG3 antibodies. While some studies have indicated that TG3 antibodies originate from cross-reactive TG2 antibodies [5], others have reported that TG2 and TG3 antibodies are not mutually cross-reactive [27,41]. Lack of cross-reactivity has been shown for both patient-derived TG2 [27] and TG3 [41] antibodies. However, the idea of separate TG2 and TG3 reactive B cells existing (as suggested above) does not explain why CeD patients do not always present with TG3 antibodies [20,43,44,45,46], and why not all CeD patients develop DH symptoms despite possessing TG3 antibodies [5,47,48]. It is known that the TG2 epitopes recognized by B cells are conformational [27], opening up the possibility of shared conformational epitopes between TG2 and TG3 when bound to different substrates. The process driving the development of high-affinity TG3 antibodies from initial low-affinity, cross-reactive TG2 antibodies is unknown and unresearched, but would most likely require repeated cycles of gluten exposure and prolonged inflammation. By assessing the degree of somatic mutations in anti-TG3 BCRs compared to anti-TG2 BCRs, it might be possible to determine whether anti-TG3 BCRs undergo affinity maturation to a higher degree than anti-TG2 BCRs. This information would be valuable in ascertaining whether anti-TG3 responses arise from anti-TG2 cross-reactivity with TG3. The previously discussed antigen availability in the intestine could also play a role in the transition from low- to high-affinity TG3 antibodies.

The current model for TG2 and TG3 antibody development suggests that anti-TG responses are T cell-dependent. B cells escape tolerance towards the autoantigens by receptor-mediated endocytosis of TG2/TG3–gliadin complexes, presenting gliadin to gliadin-specific CD4+ T cells. As for TG3 antibodies, very little research has been conducted to establish their origin. While it is possible that TG3 antibodies initially arise from strictly TG3 reactive B cells, data on CeD and DH disease progression speak against it. Due to the fact that DH is rare in children with CeD and TG3 seropositivity in CeD increases with age [20], it would seem more likely that TG3 antibody responses are somehow developmentally tied to anti-TG2 antibody responses.

### 3.2. Origins of Serum and Skin Antibodies

This section will discuss the literature available on the skin deposits of IgA and TG3 in DH, as well as the plausible sites of origin for the serum TG3 antibodies, once again using anti-TG2 antibodies in CeD as an example.

The distinguishing feature of DH is skin lesions, accompanied by closely situated deposits of IgA and TG3. These IgA–TG3 complexes are the primary diagnostic criteria for DH [46]. It is unclear where the complexes of TG3 and IgA in DH skin are formed, but it is currently assumed that they are either TG3–IgA complexes originating from the circulation [49], or IgA from circulation binding and forming complexes with TG3 in situ [50]. Complexes originating from the circulation are also supported by findings of TG3 being present in serum [15]. The complexes of TG3 and IgA are found on the dermal–epidermal boundary, where TG3 is not endogenously expressed [51]. While TG3–IgA complexes are a characteristic feature in DH, they do not seem to be pathogenic by themselves, as they are often found in areas of the skin adjacent to the actual lesions in DH [52,53], as well as occasionally also in CeD patients not exhibiting any DH symptoms [54,55,56]. As for the IgA in these complexes, very little research on the characteristics and origin is available. The scarce literature available suggests that the IgA in DH skin is in fact dimeric [57], thereby suggesting a connection with the gut. The skin-deposited antibodies in DH patients are mostly of the IgA1 subclass [58,59], like the majority of anti-TG2 IgA found in CeD patient serum [34]. Due to the paucity of literature studies on IgA-TG3 deposits in the skin, we will be focusing on the origin of serum TG3 and TG2 antibody responses for the remainder of this section.

In DH, TG3 antibody-secreting plasma cells have been found in the small intestine of patients [40,41]. Although anti-TG2 plasma cells are well-established in the gut of CeD patients [16,25,26,34,40,41], studies have found that serum TG2 antibodies and TG2 antibodies produced in the intestine have distinct molecular composition [34]. This observation opens up the possibility that individual B cell clones have given rise to distinct plasma cell populations responsible for the serum and gut antibodies [34]. Although both the gut and serum antibodies were found to target the same epitope in TG2, and had matching amino-acid sequences in the antigen-binding regions, the serum antibodies were found to be associated with less J-chain [34]. Since J-chain is the component that allows dimeric IgA to be transported into the gut lumen from the intestinal tissue, the authors hypothesized that the majority of the serum TG2 antibodies are not produced in the gut [34]. It has indeed been found that plasma cells formed during gut immune responses can contribute to the bone marrow plasma cell population, both in mice [60] and humans [34,61], and therefore it is possible that TG2 and TG3 antibodies in both DH and CeD patient serum originate from bone marrow. There is however an inconsistency in this hypothesis—the gluten dependency of serum TG2 and TG3 antibodies [18,40,62]. If serum TG2 and TG3 antibodies were produced by bone marrow plasma cells, we could expect to detect low titers regardless of GFD, as bone marrow plasma cells produce antibodies at a constant rate irrespective of antigen exposure. Both TG2 and TG3 antibody levels respond to gluten [18,40,62], with the exception of some CeD patients who experience no reduction in TG3 antibodies during GFD [20]. Regardless, due to the gluten dependency of the TG2 and TG3 antibodies [18,40,62], it is unlikely that the antibodies in patients’ sera originate from long-lived bone marrow plasma cells. In general, the functions and origins of serum IgA in humans are less well-established than those of mucosal IgA. However, it has been suggested that some of the B cells activated in gut-associated lymphoid tissues could migrate to the marginal zone in the spleen and contribute to the serum IgA pool from there [63]. This type of mechanism could explain the gluten dependency of DH and CeD TG2 and TG3 antibody responses, yet more research is required to elucidate the dynamics of humoral immune responses originating from the gut.

Little is known about the IgA–TG3 complexes in the skin, as well as serum TG3 antibodies in DH. While studies in CeD suggest that TG2 antibodies in the serum may not originate from the gut, no corresponding characterizations have been made of TG3 antibodies. However, the fact that IgA–TG3 complexes in the skin are dimeric could point towards an intestinal origin.

## 4. Conclusions

Although considerable advances have been made to ascertain how anti-TG2 responses develop in CeD, little attention has been paid to anti-TG3 immune responses and DH. Based on the literature available, we have in this review summarized what is known of the development and characteristics of anti-TG3 antibodies. Due to the lack of knowledge on anti-TG3 antibody responses in DH, we have used the literature available on anti-TG2 responses in CeD to hypothesize how anti-TG3 antibody responses might conceivably develop. The current view of anti-TG2 antibody development suggests that clonally ignorant anti-TG2 B cells are able to present gliadin to gliadin-specific CD4+ T cells in the intestine, thereby receiving activating signals. There are mainly two plausible models for the development of anti-TG3 antibody responses. These responses could either develop from TG3-specific, non-cross-reactive B cells, or they could develop from anti-TG2 antibody responses. Given that DH manifests almost exclusively in adults with CeD, it is likely that anti-TG3 responses arise from initial anti-TG2 responses, although there is little direct evidence supporting either of the two models.

More research efforts should be directed towards studying B cell responses in DH. Virtually nothing is known, for example, of the existence and longevity of TG3-linked memory cells—either of T or B cell type. Likewise, further studies should be conducted both on the expression patterns of the autoantigen TG3 and the occurrence of autoimmune-related phenomena such as the pathognomonic TG3–IgA deposits. The TG3 autoantibodies appear to originate from the gut but it is puzzling why the TG3-linked DH appears to manifest in limited areas of the skin. This is even more so, since, in the light of current knowledge, TG3 is also expressed, e.g., in the epithelium of esophagus. It is thus possible entirely possible that anti-TG3 deposit could be discovered at or near other sites of TG3 expression.
